# The Third Semi Annual Meeting of the Central States Dental Association

**Published:** 1867-07

**Authors:** 


### Proceedings of Societies.

---

### THE THIRD SEMI-ANNUAL MEETING OF THE CENTRAL STATES DENTAL ASSOCIATION.

The third semi-annual meeting of the Central States Dental Association was held in the city of Memphis, Tenn., on Tuesday, Wednesday, Thursday and Friday, the 9th, 10th, 11th and 12th of April, 1867.

The committee of arrangements and reception having procured the use of the Casina Hall, on Adams street, the Association was duly called to order at four o'clock, P. M., by the President, Dr. W. G. Redman, of Louisville.

The roll was then called, and the following members answered to their names:

W. G. Redman, *President*, Louisville, Ky.
W. H. Shadoan, *Secretary*, "
W. H. Morgan, Nashville, Tenn.
W. F. Southern, Memphis, Tenn.
Alex. Hartman, Murfreesborough, Tenn.
F. W. Garkey, Memphis, Tenn.
R. Russell, Nashville, Tenn.
G. W. Acree, Memphis, Tenn.

The constitution and by-laws were then read, after which the minutes of the last meeting were read. The society being duly organized, the executive committee made the following report, which was adopted:

### SUBJECTS FOR DISCUSSION.

1. In filling teeth, what are the points to be observed to secure success, and what are the requisite qualities of materials for filling teeth?

2. Why, and how is decay sometimes arrested without the aid of the Dentist?

3. What is the nature of sensitive dentine? its variations and treatment?

4. What principles are involved in the treatment of Dental periostitis?

5. What are the successive steps in the formation of Alveolar Abscess?

6. What is the process of absorption in living organic tissues?

7. By what means is lost tissue restored?

8. In what does a hemorrhagic diathesis consist?

9. What is the treatment of Alveolar hemorrhage?

10. Irregularities, causes, and treatment?

J. TAFT,<br>S. DRIGGS,<br>E. W. MASON, } Executive Committee.

The following applications for membership were then read by the Secretary, and referred to the committee:

Dr. L. C. Chidsholm, Tuscumbia, Ala.<br>

" J. M. Cameges, Brownsville, Tenn.<br>

" J. B. Wasson, Memphis, Tenn.<br>

" H. M. Acree, Clarksville, Tenn.<br>

" E. W. Sawyer, Memphis, Tenn.<br>

" S. H. Smith, " "<br>

" S. P. Cutler, Holly Springs, Miss.<br>

" J. C. Harris, Memphis, Tenn.<br>

" A. Wesson, " "<br>

" W. T. Arrington, Memphis, Tenn.

Adjourned to meet in Dr. Arrington's office at 7½ o'clock, P. M.

FIRST DAY—EVENING SESSION.

The Society met pursuant to adjournment. Minutes read and approved.

The committee on membership made the following report, which was adopted:

"We, the committee on membership, would respectfully recommend the following named gentlemen to the Society as eligible to membership:

L. C. Chidsholm, J. M. Cameges, H. M. Acree, E. W. Saywer, Wm. T. Arrington, S. P. Cutler and J. C. Harris.

The committee would ask further time on the following names: Drs. Smith, Wasson and Wesson, these gentlemen not being present.

G. W. ACREE,<br>W. H. MORGAN, } Committee.<br>F. W. GARKEY.

A ballot was held, and those recommended by the committee were found duly elected.

On motion, it was resolved that the sessions, during the meeting, be held as follows: From 9 A. M. to 2 P. M., and from 3 to 6 P. M. Evening sessions at 8 o'clock.

On motion of Dr. Shadoan, it was agreed that Operating Clinics be held from 11 to 1 o'clock each day. Drs. Shadoan and Arrington were appointed by the President to operate to-morrow. Drs. Russell and Hinson very magnanimously proffered themselves as subjects for the occasion.

On motion, a select committee of three was appointed by the President, consisting of Drs. Morgan, Garkey and Arrington, to examine new appliances, and report their investigations.

Adjourned to meet in this office at 9 A. M. to-morrow.

SECOND DAY—MORNING SESSION.

Minutes read and approved.

A letter was read by the Secretary from James J. Brooks, of Trenton, Tenn., which called for the following resolution:

"Be it resolved by the Central States Dental Association, that all applicants for membership must present themselves in person, when, if found eligible by the committee on membership, their names will be voted upon by the Society."

The resolution was unanimously adopted.

The first subject for consideration was then taken up and discussed.

Dr. Chidsholm—Prepares his cavities in the usual manner. Prefers soft foil, and uses it in various forms. If, in excavating a cavity, he is liable to expose the nerve, he always leaves that portion of decay just above the pulp. Believes that when a good filling is inserted, thereby excluding the fluids of the mouth, there will be no further progress of decay.

Dr. Russell—Always shapes his cavities so as to retain a filling by mechanical adaptation. Uses soft gold foil and hand pressure. Prepares his foil in cylinder form. Inserts them on end, with lateral pressure to walls, and fills to the centre. Introduces a small gold wedge or two in the central cylinders.

Dr. H. M. Acree—If confined to any one particular preparation of gold, would prefer soft foil, but thinks he can do better operating by using them alternate, as his judgment may direct. Believes in filling fangs when circumstances attending the case are favorable, but does not approve of filling the apex with cotton and creosote, as is the practice of many.

Dr. Shadoan—Uses adhesive foils exclusively, with mallet pressure. Secures a firm base and builds from the bottom up. Advocates contour fillings. If confined to any one preparation of gold, would prefer adhesive foil. Thinks an operator can serve his patrons more faithfully by the adhesive foil and mallet than in any other way. Prepares his gold in pellet cylinder form, and always passes it through a spirit flame just before using it. Advocates fang filling, and invariably uses cotton and creosote at the apex. Fills thoroughly the whole fang with the mallet, if he can. Uses the file freely.

Dr. Morgan—Makes free use of the file, and after removing the amount he thinks requisite, proceeds, with chisels and excavators, to shape his cavity in such way as to retain the filling by mechanical adjustment. Thinks it a good idea to dampen each cavity with creosote before filling. Has used carbolic acid with success in allaying sensitive dentine. Uses soft gold foil and hand pressure. Sometimes anneals his gold before using. Invariably relies upon the expansive property of his foil to secure firmness, and not its cohesiveness. Does not like adhesive foils for general use.

Dr. Arrington—In preparing his cavity uses the file freely until he secures a firm wall, after which proceeds in the usual way to prepare for filling. In proximal cavities always secures three or more retention points—two in the cervical wall, one in the coronal, and if possible, one in the lingual. Uses soft foil as a base, and builds out with annealed foil, and the mallet, whenever the case will admit. Believes there are some proximal cavities that cannot be successfully filled with the mallet. Does not believe in an exclusive mode of practice, but in combining all that is good and rejecting the bad. Advocates fang filling, and never extracts a tooth if chances are in its favor. Thinks it best always to take proper time and pains to accomplish a good operation, even if the patient is not able to pay its true value.

The hour for the Clinic having arrived, the subject under consideration was laid over, to be continued in the afternoon session.

Drs. Arrington and Shadoan, according to appointment, operated as follows: Dr. Arrington filling the left superior, second bicuspid posterior proximal surface. Dr. Snadoan, the first left superior bicuspid, anterior, proximal and fang cavity. Both using annealed gold and mallet pressure.

Adjourned to meet at Dr. Arrington's office, at 3 P. M.

### SECOND DAY—AFTERNOON SESSION.

Minutes read and approved.

The committee on membership reported the names of Drs. J. A. Arrington and S. A. Smith, who were duly elected.

The first subject was again taken up and discussed freely by several members, during which very many original, but sound ideas were suggested as to the "points to be observed to secure success," and "the requisite qualities of materials for filling teeth." Gold, however, was agreed upon as the only fit material for general use.

On motion, the Society adjourned to meet at the office of Dr. Harris, at 7½ P. M.

SECOND DAY—EVENING SESSION.

The Society met according to adjournment. Minutes read and approved.

The committee on membership reported favorable on the applications of Drs. Hinson and Wasson, whereupon, by ballot, they were duly elected.

Dr. S. P. Cutler, of Holly Springs, Miss., then exhibited, by the aid of his very powerful microscope, several interesting specimens of Dental structure, all of which he explained briefly. Dr. Cutler manufactured the instrument in his Dental office, at Holly Springs, during the war, and having obtained a magnifying power far above any microscope yet known to the profession, he hopes to throw much light upon the subject, and make satisfactory investigations upon such points as have heretofore been questioned.

Dr. William T. Arrington offered the following resolution, which was unanimously adopted: *Resolved*, that we tender a vote of thanks to Dr. Cutler for the very valuable and highly instructive information given us on the subject of microscopy. And, whereas, there are many disputed points on the subject of Dental structure, and we, the members of the Central States Dental Association, feeling ourselves highly benefited by the views put forth in the late numbers of the *Dental Cosmos*, under the head of microscopy of the teeth; therefore,

*Resolved*, That a committee of three be appointed, whose duty it shall be to furnish Dr. C. with such teeth of the various races as will enable him to prepare interesting specimens for exhibition at our next stated meeting.

Drs. W. H. SHADOAN,<br>H. M ACREE,<br>F. W. GARKEY. } *Committee.*

The meeting adjourned to meet at Dr. Garkey's office to-morrow at 9 A. M.

THIRD DAY—FORENOON SESSION.

The Association met pursuant to adjournment. Minutes read and approved.

The committee on membership presented the application of Dr. Wesson for ballot, whereupon he was elected a member of the Society.

A committee of two was appointed by the President to nominate delegates to the American Dental Association, at Cincinnati, in July next.

Drs. W. H. MORGAN, W. T. ARRINGTON, } *Committee on Nomination.*

The following resolution was then offered by Dr. Chisholm, and unanimously adopted:

Whereas, there are various forms of gold for filling teeth, and the Society having agreed that it is the best material yet known to the profession,

Be it *Resolved*, That an expression of this meeting be taken upon the use of soft or non-adhesive foils. *Second*, adhesive and non-adhesive together, *Third*, adhesive foils alone. A vote was then taken, which resulted as follows, viz:

Those who use soft foil exclusively, are Drs. Redman, Hinson, Southern and Russell. Those who use soft or adhesive gold, as circumstances indicate, are Drs. Morgan, Acree, Sawyer, Arrington, Wesson, Smith, Garkey, Harris, Chisholm, Wasson, J. A. Arrington, Cameges and Acree. The vote then being taken on adhesive foil alone, Dr. Shadoan was the only one found to advocate its exclusive use.

Recapitulation—Soft foil, 4; soft and adhesive, 13; adhesive, 1.

Dr. Shadoan's solitary vote on adhesive foil produced laughter, which called forth the expression that he would rather vote alone than with the *second class*, as he did not think that any Dentist could arrive at any great degree of perfection in filling if he is constantly changing from one kind of material to another.

A committee of publication was then appointed by the President, consisting of Drs. Arrington, Morgan and Cutler.

On motion, the eighth subject, "in what does a hemorrhagic diathesis consist," was taken up and discussed. After which the Secretary read an essay from Dr. H. S. Chase, of Iowa City, on the subject. A vote of thanks was tendered Dr. Chase, and the essay refered to the committee of publication. The subject was then dismissed and miscellaneous business taken up.

On motion of Dr. G. W. Acree, the subject of Dental ethics was taken up, and after much being said, Dr. Morgan covered the whole ground by saying that true Dental ethics consists in being an honest man and a true gentleman under any and all circumstances. The hour having arrived for clinics, a recess was had until 1 P. M.

Dr. Garkey presented two very interesting subjects to the Society from the hospital. One a re-section of two-thirds of the inferior maxillary, resulting from tertiary syphilis. The other, a polypus of antrum and nose. These two patients had been operated for a few days previous and are now doing well.

Dr. Redman's automatic condenser was exhibited in connection with some few other appliances.

At 1 o'clock the meeting was called to order, and the President appointed the following essayists:

Drs. Chisholm, H. M. Acree, S. P. Cutler, W. T. Arrington, Cameges and Sawyer.

The committee on membership reported the name of Dr. W. M. R. Johns, of Sumerville, Tenn., as an eligible person for membership, whereupon he was unanimously elected.

Dr. Morgan then offered the following resolution, which was adopted:

*Resolved*, That a committee of three be appointed by the President, whose duty it shall be to petition the Legislature to pass an act regulating the practice of Dentistry in the State of Tennessee, thereby excluding quackery. Said act or bill to be drawn up by the committee and refered to the Legislature.

Drs. Morgan, Fouche and W. T. Arrington were appointed the committee.

Dr. Hinson then exhibited an obturator made of English hard red rubber, which was perfectly flexible, and had been giving satisfaction to the patient for eight months. Articulation much improved.

Adjourned to meet at 3 P. M.

THIRD DAY—AFTERNOON SESSION.

Minutes read and approved.

The President then appointed Drs. McClellan and Canine, of Louisville, an auditing committee.

The committee on new appliances then made the following report:

Dr. W. G. Redman, of Louisville, Ky., has presented an automatic plugger, or condenser, for consolidating gold fillings, and after thoroughly investigating the merits of this instrument, invented and manufactured by Dr. Redman, *Resolved*, That it is truly effectual, and the most practical instrument of the kind yet known to the Society, and we would respectfully recommend it to the Dental profession, and suggest that the Society take steps towards its general introduction and use.

F. W. GARKEY,<br>

W. H. MORGAN,<br>

WM. T. ARRINGTON, } *Committee on appliances.*

The committee on nomination made the following report of names as delegates to the American Dental Association, to be held in Cincinnati, in July: Drs. S. P. Cutler, F. W. Garkey, Wm. T. Arrington, H. M. Acree, G. W. Acree, R. Russell, E. W. Sawyer, J. A. Arrington, Alex. Hartman, S. Hinson, W. H. Shadoan and J. Fouche. All of whom were duly elected delegates to the Association.

On motion, the Secretary then read the code of Dental Ethics adopted by the Ohio State Dental Association last summer, and proposed at the last July meeting of this Society as an amendment to the constitution. Code referred to is nearly identical with that adopted by the American Dental Association at its last meeting, in the city of Boston. No further action being taken upon the subject, as it is still pending as an amendment.

Adjourned to meet in this office at 7½ P. M.

### THIRD DAY—EVENING SESSION.

The Society met pursuant to adjournment, with all the members present.

This having been the evening set apart by the Dentists of Memphis for a social entertainment, it was explained by the President and all business postponed until to-morrow. Good music was part of the evening's entertainment, interspersed with wit and good humor. Very many appropriate toasts were drank, and ample justice done the delicious viands pre-prepared for the occasion. Adjourned at 12 o'clock, to meet at Dr. Wasson's office to-morrow at 9 A. M.

### FOURTH DAY—FORENOON SESSION.

The Socicty was called to order by the President. Minutes read and approved.

The committee on membership recommended the names of Drs. William Wasson and W. C. Willard, and upon balloting they were elected members.

Several subjects of interest were briefly discussed.

Dr. Shadoan offered the following resolution, which was adopted:

*Resolved*, That the President appoint the following committees:

First. A committee on microscopy.

Second. Why and how is decay sometimes arrested without the aid of the Dentist? What is the principle?

Third. Chemical changes produced in rubber by vulcanizing, and its probable results when brought in contact with the tissues of the mouth.

The following committees were then appointed:

| | |
|---|---|
| Drs. S. P. CUTLER,<br>J. TAFT,<br>J. C. HARRIS. | *Committee on Microscopy.* |
| Drs. J. A. ARRINGTON,<br>S. DRIGGS,<br>W. H. MORGAN. | *Committee on Decay and its arrest.* |
| Drs. F. W. GARKEY,<br>McCLELLAND,<br>COBB. | *Committee on Vulcanite Rubber.* |

The President then made a few appropriate remarks and adjourned the Society, to meet in the city of Louisville during the Christmas holidays.

W. H. SHADOAN, *Secretary.*

